# Adjuvant chemotherapy followed by concurrent chemoradiation is associated with improved survival for resected stage I‐II pancreatic cancer

**DOI:** 10.1002/cam4.1967

**Published:** 2019-01-16

**Authors:** Sung Jun Ma, Gregory M. Hermann, Kavitha M. Prezzano, Lucas M. Serra, Austin J. Iovoli, Anurag K. Singh

**Affiliations:** ^1^ Department of Radiation Medicine Roswell Park Comprehensive Cancer Center Buffalo New York; ^2^ Jacobs School of Medicine and Biomedical Sciences University at Buffalo, The State University of New York Buffalo New York

**Keywords:** adjuvant chemoradiation, adjuvant chemotherapy, adjuvant radiation, adjuvant therapy, National Cancer Database, resectable pancreatic cancer

## Abstract

**Background:**

This National Cancer Database (NCDB) analysis evaluates the clinical outcomes of postoperative chemotherapy followed by concurrent chemoradiation (C + CRT) compared to concurrent chemoradiation (CRT) alone or adjuvant chemotherapy alone (C) for resected pancreatic cancer.

**Methods:**

The NCDB was queried for primary stage I‐II, cT1‐3N0‐1M0, resected pancreatic adenocarcinoma treated with adjuvant C, CRT, or C + CRT (2004‐2015). Patients treated with C + CRT were compared with those treated with C (cohort C) and CRT (cohort CRT). Baseline patient, tumor, and treatment characteristics were examined. Kaplan‐Meier analysis, multivariable Cox proportional hazards method, forest plot, and propensity score matching were used.

**Results:**

Among 5667 patients, median follow‐up was 34.7, 45.2, and 39.7 months for the C, CRT, and C + CRT cohorts, respectively. By multivariable analysis for all patients, C and CRT had worse OS compared to C + CRT. Treatment interactions were seen among pathologically node‐positive disease. C + CRT was favored in 1‐3 and 4+ positive lymph node diseases when compared to C or CRT alone, but none of the treatment options were significantly favored in node negative disease. Using propensity score matching, 2152 patients for cohort C and 1774 patients for cohort CRT were matched. C + CRT remained significant for improved OS for both cohort C (median OS 23.3 vs 20.0 months) and cohort CRT (median OS 23.4 vs 20.8 months).

**Conclusion:**

This NCDB study using propensity score matched analysis suggests an OS benefit for C + CRT compared to C or CRT alone following surgical resection of pancreatic cancer, particularly for patients with pathologically positive lymph nodes.

## 1 INTRODUCTION

Pancreatic adenocarcinoma, the fourth leading cause of cancer death in the United States, is a treatment challenge with a dismal median survival of 12.4 months.^1^ Surgical resection is considered the only potentially curative approach, though survival rates are modest, with a 5‐year overall survival (OS) of 7%‐17%.^2^, ^3^, ^4^ With local failure rates as high as 73% after surgery,^2^, ^3^, ^4^ various adjuvant therapies, including chemoradiation (CRT), have been investigated in clinical trials and institutional studies as a means to address the poor clinical outcomes in patients with pancreatic adenocarcinoma. Several reports have demonstrated improved OS with the use of adjuvant chemoradiation, with median OS times ranging from 19.5 to 25.2 months.^5^, ^6^, ^7^, ^8^, ^9^ Several National Cancer Database (NCDB) studies have similarly shown improved OS with adjuvant CRT.^10^, ^11^, ^12^

Literature for the role of chemotherapy (C) before CRT for resected pancreatic adenocarcinoma is limited. A phase III study of adjuvant fluorouracil vs gemcitabine, given for 3 weeks followed by CRT, and then an additional 3 months of C, found no difference in OS with either agent.^13^ A NCDB analysis, did however, report a survival benefit with chemotherapy prior to CRT for locally advanced pancreatic cancer.^14^ Due to a lack of comparative studies, the value of C prior to CRT specifically for early‐stage pancreatic cancer remains unclear.

This study compares the outcomes of patients who received C + CRT vs those who received C or CRT alone for stage I‐II, resected pancreatic cancer.

## 2 METHODS

### 2.1 Patient population

The NCDB registry was used to identify patients with pancreatic adenocarcinoma diagnosed between 2004 and 2015 (the most recent dataset available at the time of this study). The NCDB is a nationwide cancer database that captures approximately 70% of newly diagnosed cancer cases in the United States and includes 34 million historical records.^15^ It provides access to de‐identified datasets from Commission on Cancer‐accredited programs through online application. This study was exempt from institutional review board review.

**Figure 1:**
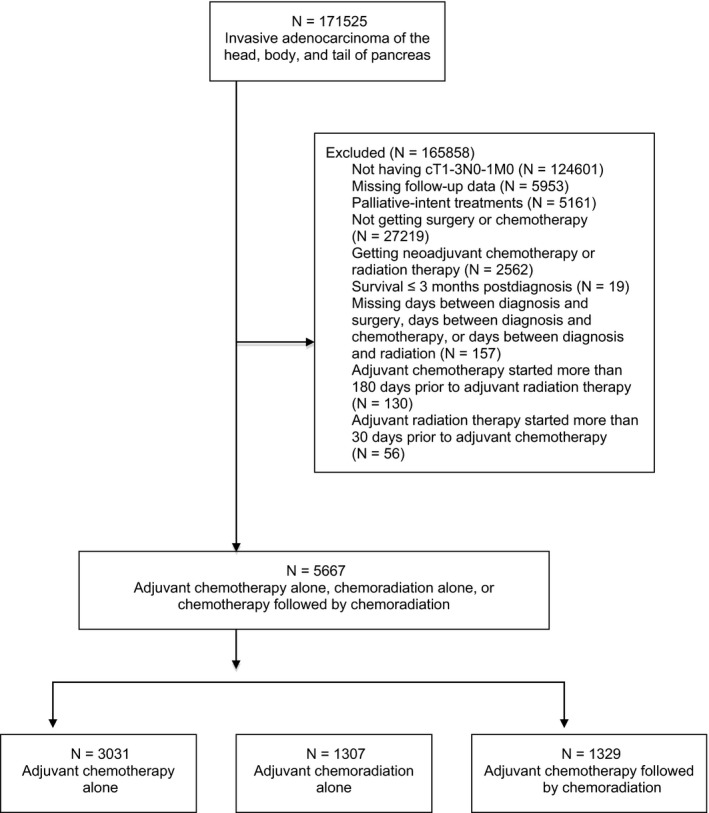
CONSORT diagram for patient selection criteria

Our patient selection criteria are shown in Figure 1. We selected from our initial query of patients with stage I‐II, clinical T1‐3N0‐1M0 pancreatic adenocarcinoma who had been treated with curative‐intent resection followed by adjuvant chemotherapy and conventionally fractionated radiation therapy. American Joint Committee on Cancer (AJCC) 6th and 7th editions were used to determine stage I‐II disease in 2004‐2015.

Whipple surgery was defined as local or partial pancreatectomy and duodenectomy with partial gastrectomy. Whipple‐variant surgery was characterized as partial pancreatectomy with duodenectomy, total pancreatectomy alone, or total pancreatectomy with subtotal gastrectomy or duodenectomy.^16^ Patients treated with chemotherapy or radiation therapy within 30 days of each other were considered to have received adjuvant CRT alone. Those who were treated with adjuvant chemotherapy within 31‐180 days prior to the radiation therapy were defined as having received C + CRT.^14^ Patients who received adjuvant chemotherapy more than 180 days prior to adjuvant radiation therapy were excluded.

Patients were excluded if they had incomplete follow‐up data, missing radiation dose or fractionation information, incomplete data on the number of days between diagnosis and treatments, or missing information regarding surgical margins. Patients treated with palliative‐intent or with neoadjuvant chemotherapy or radiation were also excluded. To address immortal time bias, those with postdiagnosis survival duration of <3 months were not included.^17^

Baseline patient, tumor, and treatment characteristics for analysis included the following: facility type, age, gender, race, insurance type, income level, residential setting, Charlson‐Deyo comorbidity score (CDS), year of diagnosis, primary tumor location within pancreas, tumor grade, tumor size, clinical T and N stages, pathologic T and N stages, number of biopsy‐positive lymph nodes, surgery type, surgical margin, total radiation dose, and chemotherapy use. Surgical margin was categorized as either negative (R0) or positive (R1, R2, positive margin not otherwise specified). Patients were stratified by age ≥66 or <66 years, and tumor size <3.1 or ≥3.1 cm based on their median values. The household income level of each patient's residential area was based on the 2012 American Community Survey data adjusted for inflation (the most recent data at the time of this study) and was stratified above or below the median value of $48 000. CA 19‐9 factor was coded by the NCDB with a cut off of <98 or ≥98 Units/mL, although CA 19‐9 was not used for propensity score matching due to missing data in 3064 (54.1%) of patients. Local and distant failure/progression information is also unable to be analyzed based on data from the NCDB. Important prognostic variables such as the patient's initial performance status, type and duration of chemotherapy received, and toxicity outcomes are unavailable in the NCDB. The primary endpoint was overall survival (OS), time between the diagnosis and the last follow‐up or death.

### 2.2 Statistical analysis

OS was evaluated using the Kaplan‐Meier method and log‐rank tests. Fisher's exact test and Mann‐Whitney *U* test were used to compare categorical and continuous variables between two treatment cohorts, respectively. Logistic regression univariable (UVA) and multivariable analyses (MVA) were used to determine potential factors that predicted the use of postoperative chemotherapy and were reported as odds ratio (OR). Cox proportional hazard UVA and MVA were used to determine factors that predict the OS and were reported as hazards ratio (HR). MVA was initially constructed using all statistically significant variables from UVA and was finalized using a backward stepwise elimination. Only patients with complete information on such variables were included. Potential interactions between the treatment and other covariates were examined using Cox MVA by adding interaction terms.^18^ When the interaction terms were statistically significant, the final Cox MVA model was re‐analyzed for each subgroup of covariates, and a forest plot was constructed to illustrate the direction and magnitude of treatment effects.^18^

To minimize selection bias, propensity score matching was used. Match‐pairs were constructed by matching baseline patient, tumor, and treatment characteristics. Variables of interest include facility type, year of diagnosis, age, CDS, tumor grade, tumor size, surgery type, chemotherapy use, total radiation dose, pathologic T and N stages, and additional variables that were statistically significant in Cox proportional hazard MVA results for each cohort. All matching was performed in a 1:1 ratio without any replacement and was based on nearest neighbor method with a caliper distance of 0.2 of the standard deviation of the logit of the propensity score.^19^ Matching was performed using MatchIt package (version 3.0.1). R software (version 3.5.0, R Foundation for Statistical Computing, Vienna, Austria) was used for all aforementioned analyses. All *P* values were two‐sided. A *P* value <0.05 was considered statistically significant.

## 3 RESULTS

A total of 5667 patients with resected clinical stage I‐II, T1‐3N0‐1M0 pancreatic adenocarcinoma were identified for analysis. Of those, adjuvant C, CRT, and C + CRT were delivered to 3031, 1307, and 1329 patients, respectively. Overall follow‐up was 37.4 months (IQR [interquartile range] 24.5‐59.3). The majority of patients had pathologic T3N1 adenocarcinoma of the pancreatic head with negative surgical margins (Tables 1 and 2). Of the 2636 (1307 + 1329) patients who received RT, 2050 (1001 for CRT, 1049 for C + CRT) patients received RT to the pancreas, and 420 (221 for CRT, 199 for C + CRT) patients received RT to the abdomen (not otherwise specified); therefore, 93.7% (2470/2636) of patients received RT to the pancreas or abdomen. A total of 107 (107/1307 = 8.2%) and 106 (106/1329 = 8.0%) patients received <45 Gy in cohort CRT and C + CRT, respectively.

**Table 1:** Baseline characteristics for cohort C

	Before matching					After matching				
	C		C + CRT		*P*	C		C + CRT		*P*
	N	%	N	%		N	%	N	%	
Facility										
Nonacademic	1454	48	789	59	<0.001	622	58	631	59	0.73
Academic	1557	51	521	39		454	42	445	41	
NA	20	1	19	1		0	0	0	0	
Age										
<66	1297	43	762	57	<0.001	620	58	589	55	0.19
≥66	1734	57	567	43		456	42	487	45	
NA	0	0	0	0		0	0	0	0	
Gender										
Female	1497	49	663	50	0.77					
Male	1534	51	666	50						
NA	0	0	0	0						
Race										
White	2645	87	1148	86	0.60					
Black	270	9	121	9						
Other	95	3	49	4						
NA	21	1	11	1						
Insurance										
None	70	2	34	3	<0.001					
Nonprivate	1846	61	684	51						
Private	1096	36	601	45						
NA	19	1	10	1						
Income										
Above median	1947	64	870	65	0.37	704	65	707	66	0.93
Below median	1053	35	441	33		372	35	369	34	
NA	31	1	18	1		0	0	0	0	
Residential setting										
Metro	2470	81	1086	82	0.84					
Urban	410	14	181	14						
Rural	49	2	18	1						
NA	102	3	44	3						
Charlson‐Deyo Score										
0‐1	2805	93	1248	94	0.11	1011	94	1008	94	0.86
≥2	226	7	81	6		65	6	68	6	
NA	0	0	0	0		0	0	0	0	
Year of diagnosis										
2004‐2007	195	6	74	6	<0.001	62	6	46	4	0.13
2008‐2011	1376	45	696	52		506	47	543	50	
2012‐2015	1460	48	559	42		508	47	487	45	
NA	0	0	0	0		0	0	0	0	
Primary tumor site										
Head	2391	79	1105	83	<0.001					
Body	237	8	105	8						
Tail	403	13	119	9						
NA	0	0	0	0						
Tumor grade										
Well diff	202	7	112	8	0.024	78	7	91	8	0.083
Mod diff	1450	48	652	49		559	52	566	53	
Poor diff	1126	37	448	34		431	40	400	37	
Other	30	1	20	2		8	1	19	2	
NA	223	7	97	7		0	0	0	0	
Tumor size (cm)										
<3.1	1466	48	657	49	0.35	526	49	526	49	1
≥3.1	1511	50	636	48		550	51	550	51	
NA	54	2	36	3		0	0	0	0	
Clinical T stage										
1	505	17	195	15	0.15					
2	1180	39	509	38						
3	1346	44	625	47						
NA	0	0	0	0						
Clinical N stage										
0	2122	70	871	66	0.0036					
1	909	30	458	34						
NA	0	0	0	0						
Pathologic T stage										
0	1	0	1	0	0.013	0	0	1	0	0.81
1	158	5	45	3		41	4	37	3	
2	404	13	153	12		110	10	123	11	
3	2299	76	1056	79		908	84	898	83	
4	38	1	22	2		17	2	17	2	
Other	1	0	0	0		0	0	0	0	
NA	130	4	52	4		0	0	0	0	
Pathologic N stage										
0	900	30	293	22	<0.001	257	24	256	24	1
1	1988	66	971	73		819	76	820	76	
NA	143	5	65	5		0	0	0	0	
Number of positive lymph nodes										
0	920	30	297	22	<0.001					
1‐3	1173	39	585	44						
4+	848	28	415	31						
NA	90	3	32	2						
Surgery										
Whipple variant	941	31	397	30	0.33	364	34	325	30	0.079
Whipple	1409	46	650	49		477	44	528	49	
Other	681	22	282	21		235	22	223	21	
NA	0	0	0	0		0	0	0	0	
Surgical margin										
Negative	2429	80	968	73	<0.001	826	77	807	75	0.36
Positive	510	17	336	25		250	23	269	25	
NA	92	3	25	2		0	0	0	0	
Chemotherapy										
Single agent	2376	78	766	58	<0.001	650	60	634	59	0.51
Multi agent	655	22	563	42		426	40	442	41	
NA	0	0	0	0		0	0	0	0	
Radiation dose (Gy)										
Median	—		50.4		NA					
IQR	—		50.0‐50.4							

C, chemotherapy; CRT, chemoradiation; diff, differentiated; IQR, interquartile range; mod, moderately; NA, not available; poor, poorly.

**Table 2:** Baseline characteristics for cohort CRT

	Before matching					After matching				
	CRT		C + CRT		*P*	CRT		C + CRT		*P*
	N	%	N	%		N	%	N	%	
Facility										
Nonacademic	848	65	789	59	0.0085	548	62	539	61	0.70
Academic	452	35	521	39		339	38	348	39	
NA	7	1	19	1		0	0	0	0	
Age										
<66	674	52	762	57	0.0030	472	53	498	56	0.23
≥66	633	48	567	43		415	47	389	44	
NA	0	0	0	0		0	0	0	0	
Gender										
Female	645	49	663	50	0.79					
Male	662	51	666	50						
NA	0	0	0	0						
Race										
White	1141	87	1148	86	0.19					
Black	119	9	121	9						
Other	32	2	49	4						
NA	15	1	11	1						
Insurance										
None	31	2	34	3	0.071					
Nonprivate	728	56	684	51						
Private	533	41	601	45						
NA	15	1	10	1						
Income										
Above median	733	56	870	65	<0.001	547	62	563	63	0.46
Below median	544	42	441	33		340	38	324	37	
NA	30	2	18	1		0	0	0	0	
Residential setting										
Metro	1023	78	1086	82	0.049					
Urban	201	15	181	14						
Rural	31	2	18	1						
NA	52	4	44	3						
Charlson‐Deyo Score										
0‐1	1235	94	1248	94	0.56	836	94	841	95	0.68
≥2	72	6	81	6		51	6	46	5	
NA	0	0	0	0		0	0	0	0	
Year of diagnosis										
2004‐2007	210	16	74	6	<0.001	69	8	47	5	0.11
2008‐2011	680	52	696	52		458	52	467	53	
2012‐2015	417	32	559	42		360	41	373	42	
NA	0	0	0	0		0	0	0	0	
Primary tumor site										
Head	1076	82	1105	83	0.015					
Body	78	6	105	8						
Tail	153	12	119	9						
NA	0	0	0	0						
Tumor grade										
Well diff	108	8	112	8	0.64	75	8	79	9	0.13
Mod diff	629	48	652	49		460	52	478	54	
Poor diff	455	35	448	34		346	39	315	36	
Other	13	1	20	2		6	1	15	2	
NA	102	8	97	7		0	0	0	0	
Tumor size (cm)										
<3.1	575	44	657	49	0.0050	410	46	410	46	1
≥3.1	697	53	636	48		477	54	477	54	
NA	35	3	36	3		0	0	0	0	
Clinical T stage										
1	158	12	195	15	0.15					
2	517	40	509	38						
3	632	48	625	47						
NA	0	0	0	0						
Clinical N stage										
0	874	67	871	66	0.48					
1	433	33	458	34						
NA	0	0	0	0						
Pathologic T stage										
0	1	0	1	0	0.61	1	0	1	0	0.95
1	49	4	45	3		30	3	36	4	
2	169	13	153	12		108	12	105	12	
3	980	75	1056	79		731	82	729	82	
4	22	2	22	2		17	2	16	2	
Other	0	0	0	0		0	0	0	0	
NA	86	7	52	4		0	0	0	0	
Pathologic N stage										
0	338	26	293	22	0.0075	228	26	226	25	0.96
1	874	67	971	73		659	74	661	75	
NA	95	7	65	5		0	0	0	0	
Number of positive lymph nodes										
0	363	28	297	22	<0.001					
1‐3	585	45	585	44						
4+	324	25	415	31						
NA	35	3	32	2						
Surgery										
Whipple variant	342	26	397	30	0.10	253	29	256	29	0.98
Whipple	668	51	650	49		437	49	436	49	
Other	297	23	282	21		197	22	195	22	
NA	0	0	0	0		0	0	0	0	
Surgical margin										
Negative	891	68	968	73	0.012	643	72	637	72	0.79
Positive	386	30	336	25		244	28	250	28	
NA	30	2	25	2		0	0	0	0	
Chemotherapy										
Single agent	824	63	766	58	0.0047	536	60	515	58	0.33
Multi agent	483	37	563	42		351	40	372	42	
NA	0	0	0	0		0	0	0	0	
Radiation dose (Gy)										
Median	50.4		50.4		0.18	50.4		50.4		0.17
IQR	50.0‐54.0		50.0‐50.4			50.0‐54.0		50.0‐50.4		

C, chemotherapy; CRT, chemoradiation; diff, differentiated; IQR, interquartile range; mod, moderately; NA, not available; poor, poorly.

On logistic MVA for all patients, patients with diagnosis between 2008 and 2011 (OR 2.17, *P* < 0.001) and 2012 and 2015 (OR 1.99, *P* < 0.001), pathologic nodal diseases (OR 1.37, *P* < 0.001 for 1‐3 positive nodes; OR 1.32, *P* = 0.0034 for 4+ positive nodes), positive surgical margin (OR 1.25, *P* = 0.0062), and receipt of multiagent chemotherapy (OR 2.01, *P* < 0.001) were more likely to receive C + CRT compared to C or CRT alone. Patients treated at academic facilities (OR 0.73, *P* < 0.001), older than 66 years old (OR 0.66, *P* < 0.001), from low‐income regions (OR 0.83, *P* = 0.0083), with pancreatic tail disease (OR 0.68, *P* = 0.0011), and poorly differentiated histology (OR 0.74, *P* = 0.019) were less likely to undergo C + CRT.

On Cox MVA for all patients (Table 3), those older than 66 years old (HR 1.14, *P* < 0.001), from low‐income regions (HR 1.10, *P* = 0.0082), with higher CDS (HR 1.23, *P* = 0.0017), moderately (HR 1.18, *P* = 0.018) or poorly differentiated (HR 1.51, *P* < 0.001) disease, tumors larger than 3.1 cm (HR 1.26, *P* < 0.001), pathologic positive nodal diseases (HR 1.46, *P* < 0.001 for 1‐3 positive nodes; HR 1.79, *P* < 0.001 for 4+ positive nodes), high CA 19‐9 (≥98 U/mL) (HR 1.30, *P* < 0.001), and positive surgical margins (HR 1.47, *P* < 0.001) were associated with worse mortality. When compared to C + CRT, those treated with C (HR 1.31, *P* < 0.001) or CRT alone (HR 1.24, *P* < 0.001) had worse survival outcomes. Improved overall survival was observed in those treated at academic facilities (HR 0.83, *P* < 0.001) and pathologic T1‐2 diseases (HR 0.87, *P* = 0.0051).

**Table 3:** Cox UVA and MVA for all cohorts

Variable	Cox UVA			Cox MVA		
	HR	95% CI	*P*	HR	95% CI	*P*
Facility						
Nonacademic	1	Ref		1	Ref	
Academic	0.88	0.82‐0.93	<0.001	0.83	0.78‐0.89	<0.001
Age						
<66	1	Ref		1	Ref	
≥66	1.16	1.09‐1.23	<0.001	1.14	1.06‐1.22	<0.001
Gender						
Female	1	Ref				
Male	1.01	0.95‐1.08	0.64			
Race						
White	1	Ref				
Black	0.98	0.88‐1.10	0.76			
Other	0.95	0.79‐1.14	0.55			
Insurance						
None	1	Ref				
Nonprivate	1.15	0.94‐1.42	0.18			
Private	0.95	0.77‐1.17	0.64			
Income						
Above median	1	Ref		1	Ref	
Below median	1.14	1.07‐1.21	<0.001	1.10	1.02‐1.18	0.0082
Residential setting						
Metro	1	Ref		1	Ref	
Urban	1.08	0.99‐1.18	0.086			
Rural	1.37	1.10‐1.71	0.0052	1.20	0.94‐1.53	0.15
Charlson‐Deyo score						
0‐1	1	Ref		1	Ref	
≥2	1.2	1.06‐1.35	0.0033	1.23	1.08‐1.40	0.0017
Year of diagnosis						
2004‐2007	1	Ref				
2008‐2011	0.97	0.88‐1.08	0.63			
2012‐2015	0.90	0.80‐1.00	0.051			
Primary tumor site						
Head	1	Ref				
Body	0.92	0.81‐1.03	0.15			
Tail	0.95	0.87‐1.05	0.33			
Tumor grade						
Well diff	1	Ref		1	Ref	
Mod diff	1.22	1.07‐1.38	0.0022	1.18	1.03‐1.35	0.018
Poor diff	1.60	1.41‐1.82	<0.001	1.51	1.32‐1.73	<0.001
Other	1.19	0.86‐1.66	0.30			
Tumor size (cm)						
<3.1	1	Ref		1	Ref	
≥3.1	1.42	1.34‐1.52	<0.001	1.26	1.18‐1.35	<0.001
Pathologic T stage						
0‐2	1	Ref		1	Ref	
3‐4	0.72	0.66‐0.79	<0.001	0.87	0.79‐0.96	0.0051
Number of positive lymph nodes						
0	1	Ref		1	Ref	
1‐3	1.61	1.48‐1.74	<0.001	1.46	1.34‐1.59	<0.001
4+	2.08	1.91‐2.27	<0.001	1.79	1.63‐1.97	<0.001
Surgery						
Whipple variant	1	Ref				
Whipple	1.04	0.97‐1.12	0.30			
Other	1.03	0.94‐1.12	0.51			
Surgical margin						
Negative	1	Ref		1	Ref	
Positive	1.64	1.53‐1.76	<0.001	1.47	1.36‐1.59	<0.001
Chemotherapy						
Single agent	1	Ref				
Multi agent	1.03	0.96‐1.10	0.47			
Radiation dose (Gy)						
1 Gy increase	1.00	1.00‐1.00	1			
Treatment						
C + CRT	1	Ref		1	Ref	
CRT	1.19	1.09‐1.30	<0.001	1.24	1.12‐1.37	<0.001
C	1.15	1.07‐1.24	<0.001	1.31	1.20‐1.43	<0.001

CI, confidence interval; diff, differentiated; HR, hazard ratio; mod, moderately; MVA, multivariable analysis; poor, poorly; Ref, reference; UVA, univariate analysis.

**Figure 2:**
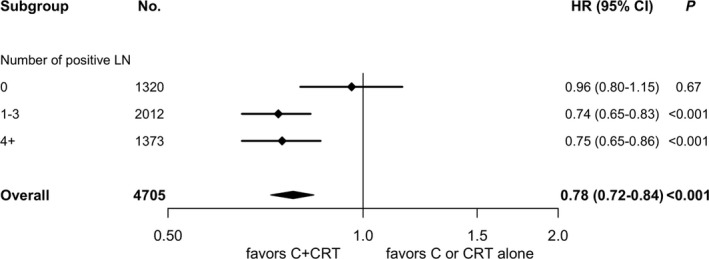
Forest plot for subgroup analysis. C, adjuvant chemotherapy; C + CRT, adjuvant chemotherapy followed by chemoradiation; CI, confidence interval; CRT, chemoradiation; HR, hazards ratio; LN, lymph node; No., number of patients

After Cox MVA, treatment interactions were observed in positive nodal disease subgroups (1‐3 positive nodes: HR 0.78, *P* = 0.020; 4+ positive nodes: HR 0.79, *P* = 0.041). No other treatment interactions were seen in age (HR 0.94, *P* = 0.46), CDS (HR 0.98, *P* = 0.88), years of diagnosis (2008‐2011: HR 1.05, *P* = 0.76; 2012‐2015: HR 1.05, *P* = 0.79), tumor size (HR 0.93, *P* = 0.40), surgical margin (HR 0.89, *P* = 0.23), or pathologic T stages (HR 1.10, *P* = 0.40). On subgroup analysis (Figure 2), nodal disease favored C + CRT when compared to C or CRT alone (0 positive node: HR 0.96, *P* = 0.67; 1‐3 positive nodes: HR 0.74, *P* < 0.001; 4+ positive nodes: HR 0.75, *P* < 0.001).

### 3.1 Cohort C

The C group had a median follow‐up of 34.7 months (IQR 22.9‐54.6), and the C + CRT group had that of 39.7 months (IQR 26.7‐59.5). The median OS was 21.1 months (IQR 12.0‐34.7) for the C group and 23.4 months (IQR 15.6‐39.3) for the C + CRT group (log‐rank *P* < 0.001). OS at 2 years was 48.8% for the C group and 53.1% for the C + CRT group.

**Figure 3:**
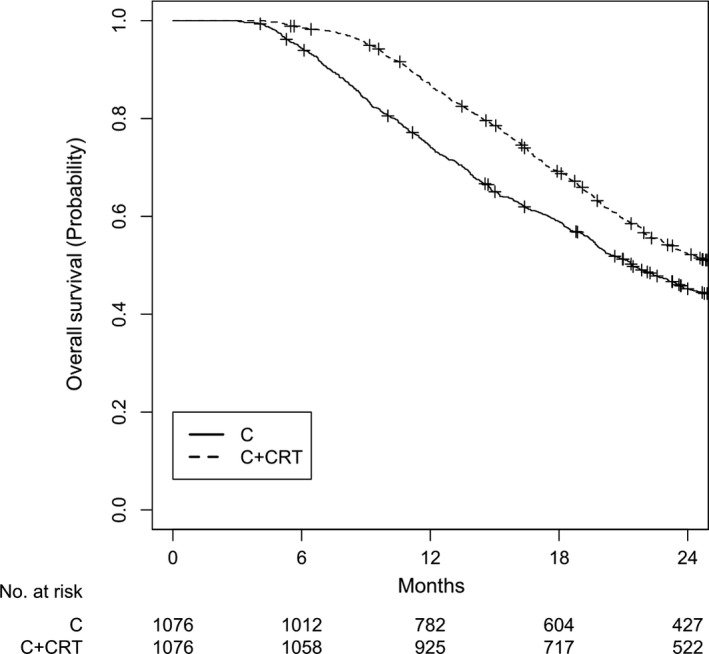
Overall survival for cohort C after matching. *P* < 0.001. C, adjuvant chemotherapy; C + CRT, adjuvant chemotherapy followed by chemoradiation

A total of 2152 patients were matched. All variables were well balanced between these two groups (Table 1). The overall median follow‐up for the matched patients was 36.7 months (IQR 24.7‐54.5). The median OS was 20.0 months (IQR 11.5‐33.6) for the C group and 23.3 months (IQR 15.6‐39.2) for the C + CRT group (Figure 3; log‐rank *P* < 0.001). OS at 2 years was 45.2% for the C group and 52.3% for the C + CRT group.

### 3.2 Cohort CRT

The CRT and C + CRT groups had a median follow‐up of 45.2 and 39.7 months, respectively. The median OS was 21.1 months (IQR 12.5‐36.0) for the CRT group and 23.4 months (15.6‐39.3) for the C + CRT group (log‐rank *P* < 0.001). OS at 2 years was 46.2% and 53.1% for the CRT and C + CRT groups, respectively.

**Figure 4:**
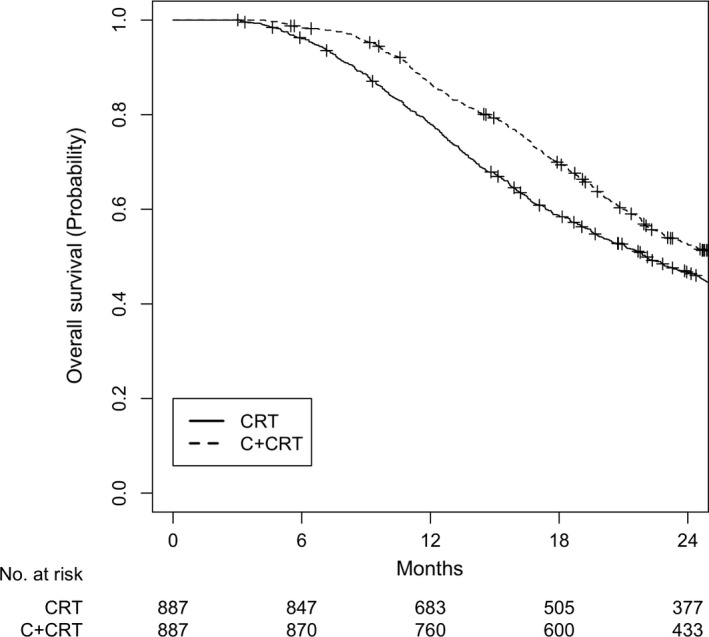
Overall survival for cohort CRT after matching. *P* < 0.001. CRT, adjuvant chemoradiation; C + CRT, adjuvant chemotherapy followed by chemoradiation

A total of 1774 patients were matched. All variables were well balanced (Table 2). The overall follow‐up was 40.2 months (IQR 26.0‐58.3). The CRT group had a median OS of 20.8 months (IQR 12.5‐34.7) and the C + CRT group had that of 23.4 months (IQR 16.0‐40.0). OS at 2 years was 46.6% for the CRT group and 52.5% for the C + CRT group (Figure 4; log‐rank *P* < 0.001).

## 4 DISCUSSION

To our knowledge, this is the first study to compare adjuvant C + CRT vs C or CRT alone for stage I‐II resected pancreatic cancer. This study suggests a survival benefit favoring the use of C + CRT for resected pancreatic cancer, specifically in cases of pathologically node‐positive disease.

The C + CRT cohort included over 70% patients with pathologically staged T3 and N1 disease, which are notably associated with worse prognosis.^20^, ^21^, ^22^ Despite this, the C + CRT still had better OS compared to the CRT alone cohort. The median OS was 23 months, which is comparable to or better than previously reported survival outcomes for adjuvant CRT alone.^5^, ^6^, ^7^, ^8^, ^9^, ^10^

The use of adjuvant C in addition to CRT has only been investigated in a few studies.^6^, ^23^, ^24^, ^25^ RTOG 9704 delivered C before and after adjuvant CRT for resected pancreatic cancer. A large number of included patients had T3‐4N1 disease and positive surgical margins. Median OS was 17.1 months for fluorouracil and 20.5 months for gemcitabine.^25^ Likely due to the initial publication of RTOG 9704 in 2008, our logistic MVA results demonstrated that those diagnosed between 2008 and 2015 were more likely to receive C + CRT compared to those diagnosed between 2004 and 2007. A prior institutional study showed that delaying CRT until after >1 cycle of adjuvant C is not associated with worse mortality when compared to adjuvant CRT only.^24^ Our study is the first report showing that delaying CRT until after 30‐180 days of adjuvant C may have survival benefits.

Our Cox MVA results (Table 3) showed that moderately or poorly differentiated tumors, larger tumor size, and pathologic N1 disease were adverse prognostic factors for mortality. This association is consistent with prior studies.^26^, ^27^, ^28^, ^29^, ^30^, ^31^, ^32^, ^33^, ^34^, ^35^ Older age, more medical comorbidities, low income, and positive surgical margins were also shown to be associated with worse mortality in our study, and this finding is also consistent with other reports.^36^, ^37^, ^38^, ^39^ In this study, treatment at academic facilities was an independent favorable prognostic factor for OS. This finding is consistent with prior analyses showing improved outcomes at high volume centers related to better surgical outcomes, which may explain why living in a rural area was associated with worse mortality.^40^, ^41^, ^42^ Although additional factors contributing to improved OS at academic facility may include patient self‐selection, higher socioeconomic status and/or performance status.

From our logistic MVA results, patients with pathologic T3‐4N1 diseases and positive surgical margins were more likely to receive C + CRT. A prior study has also shown that patients with a higher disease burden were more likely to receive adjuvant therapies.^43^

In our study, the use of multiagent chemotherapy was not a favorable prognostic factor for survival. This finding is in contrast to theEuropean Study Group for Pancreatic Cancer‐4 (ESPAC‐4) trial. Despite including 61% of patients with positive surgical margins and 79% with N1 disease in the ESPAC‐4 trial, adjuvant gemcitabine combined with capecitabine significantly improved survival.^28^ In NCDB, multiagent chemotherapy was recorded as the first course, and it is possible that some chemotherapy regimens were changed during the course of treatments. This change in chemotherapy regimens is not recorded in NCDB, which may explain this discrepancy.

### 4.1 Limitations

This study has a number of limitations, many of which are inherent to performing a retrospective review. Various potential prognostic factors, such as smoking and alcohol history, performance status, molecular tests, and the type and duration of chemotherapy, are not recorded by the NCDB. Outcomes such as local or distant recurrences, toxicity, and cancer‐specific survival were also unavailable. More than half of the CA 19‐9 values, an important prognostic factor for resectability and survival, were missing from this dataset and could not be included for propensity score matched analysis.^27^, ^44^, ^45^, ^46^, ^47^ The NCDB also does not include information on disease progression; therefore, this study cannot address the possibility of a patient received RT for progression of disease on chemotherapy. This limitation is inherent to all NCDB analyses and limit interpretation of our findings. Further, RT may have been palliative‐intent for a minority of the included patients based on site and dose of RT, but since both CRT and C‐CRT had similar numbers of patients receiving <45 Gy, it is unlikely these patients would change the conclusions of this study. Since the NCDB is not a population‐based database, our findings may not be generalized to other patient populations.

Up to 79% of patients in RTOG 9704 experienced chemotherapy‐related toxicity. A meta‐analysis of adjuvant treatments for resected pancreatic adenocarcinoma also showed significant toxicity with the addition of chemotherapy to chemoradiation.^48^ It is possible that those patients who received C + CRT may have had a better initial performance status in order to tolerate the additional toxicity of chemotherapy, thus leading to better survival outcomes compared to those receiving C or CRT alone.^13^ This potential confounder may also explain the improved survival seen in other institutional studies.^6^, ^23^ However, it is unlikely that improved performance status was the only factor contributing to this overall survival benefit, since patients with node negative diseases would have also favored C + CRT in our study. In addition, no treatment interaction was seen with CDS or with age on Cox MVA in this study, nor were CDS or age predictors on logistic MVA for the receipt of C + CRT. Since all patients in our study underwent adjuvant therapies, the difference in performance status between the C + CRT and other regimens is unlikely to independently explain the survival benefits.

## 5 CONCLUSION

In summary, this analysis suggests improved survival for adjuvant C + CRT following resected pancreatic cancer with node‐positive disease. More studies may be warranted to investigate the benefit of adding adjuvant chemotherapy to CRT and the ideal sequencing of these regimens.

## CONFLICT OF INTEREST

All authors declare that they have no competing interests.
